# Great Toe Infection Leading to Methicillin-Sensitive Staphylococcus aureus Pericarditis and Tamponade

**DOI:** 10.7759/cureus.32951

**Published:** 2022-12-26

**Authors:** Devin C Weber, Rana Wajahat

**Affiliations:** 1 Infectious Diseases, University Hospitals Portage Medical Center, Ravenna, USA; 2 Infectious Diseases, Lincoln Memorial University DeBusk College of Osteopathic Medicine, Harrogate, USA; 3 Infectious Diseases, Western Reserve Hospital, Cuyahoga Falls, USA

**Keywords:** septic shock, pericardial effusion, toe amputation, pericardiocentesis, cardiac tamponade, mssa pericarditis, bacterial pericarditis, purulent pericarditis

## Abstract

Bacterial pericarditis is an uncommon presentation that can occur secondarily to the contiguous spread of infection from an intrathoracic focus, hematogenous seeding from a distant site, or via trauma and intrathoracic surgery. Its presence is linked to a high mortality rate with death generally caused by cardiac tamponade, fulminant sepsis, and acute decompensated heart failure. We describe a rare case of methicillin-sensitive Staphylococcus aureus (MSSA) pericarditis in a patient after great toe amputation; the patient developed cardiac tamponade and required urgent percutaneous pericardiocentesis with the placement of a temporary drain. The patient was then successfully treated with IV antibiotics and did not require further invasive procedures such as surgical pericardiotomy.

## Introduction

Pericardial effusion, either found incidentally on chest CT or echocardiography, or in connection with a systemic or cardiac disease, is not an uncommon finding in clinical practice [1]. There are numerous causes of pericardial effusions; they are generally divided into inflammatory and non-inflammatory etiologies [2]. Common causes of pericardial effusions include infections (viral, bacterial, and rarely tuberculosis), Dressler syndrome, malignancy, autoimmune disorders, and diseases of the myopericardium and aorta [1]. However, purulent bacterial infections within the pericardial space are an uncommon cause of pericarditis or effusion [3].

Bacterial pericarditis is less prevalent in the present antibiotic era and the overwhelming majority of cases occur in those who are immunocompromised or in individuals with an underlying disease of the pericardium [4,5]. This disease process occurs via many mechanisms including hematogenous dissemination, contiguous spread from an intrathoracic, myocardial, or subdiaphragmatic focus, or direct infection during trauma, thoracic surgery, or catheter drainage [6]. It is associated with a high mortality rate given its ability to rapidly progress to tamponade and death [7]. Here, we present a case of bacterial pericarditis, days after great toe amputation, resulting in cardiac tamponade which was successfully managed with pericardial drainage and IV antibiotics. 

## Case presentation

A 79-year-old Caucasian male presented to the hospital with underlying medical problems; his medical history was significant for coronary artery disease status post coronary artery bypass grafting (CABG), peripheral vascular disease status post bilateral lower extremity stenting, hypertension, hyperlipidemia, gastroesophageal reflux disease (GERD), well-controlled non-insulin dependent type 2 diabetes mellitus (A1c 7.2% on hospitalization), anxiety, insomnia, and a recurrent right great toe infection. He reported a one-day history of progressively worsening generalized weakness, lethargy, and increased bilateral lower extremity heaviness. He stated that his symptoms worsened as the day progressed and began experiencing dyspnea at rest upon presenting to his regularly scheduled cardiology appointment for a two-month follow-up of a right superficial femoral artery percutaneous transluminal angioplasty. After the initial assessment, his cardiologist recommended further evaluation and treatment at the emergency department.

At the emergency room, his initial vitals were as follows: temperature of 36.5 Celsius, heart rate of 81 bpm, respiratory rate of 18, blood pressure of 83/47 mm Hg, and pulse oxygen of 97% on 3L via nasal cannula. The initial ECG showed concerns for ST-elevation myocardial infarction (STEMI), most prominent in the inferior leads. Therefore, STEMI protocol was activated and the patient was given aspirin, and ticagrelor was loaded. The cardiologist who was consulted did not think it was STEMI and considered other etiologies such as underlying renal dysfunction and lower extremity infection with septic shock. Despite IV fluid resuscitation, he remained hypotensive requiring vasopressor support. After blood cultures were drawn, the patient was started on registered pharmacist (RPh) dosed IV vancomycin and 3.375g IV piperacillin-tazobactam.

**Figure 1:**
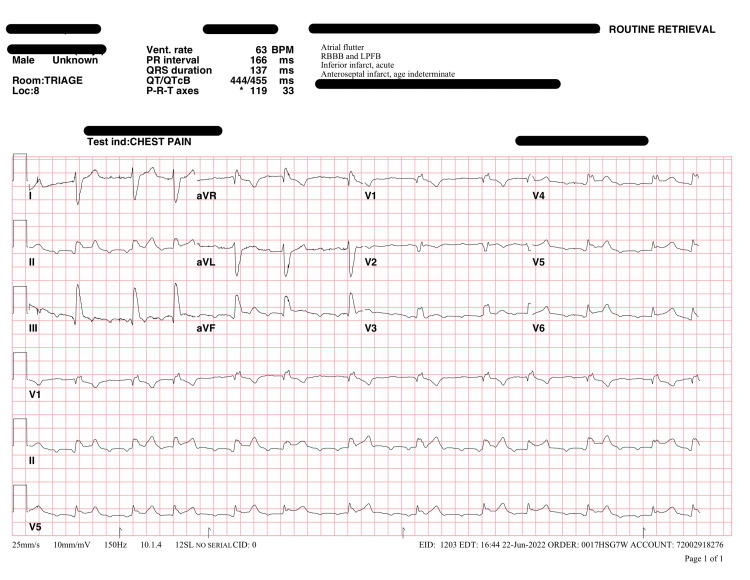
Initial ECG showing atrial flutter and variable block with ST elevation in lead II and to a lesser extent in aVF/V5/V6 without reciprocal changes. Serial ECGs in the emergency department showed resolution of the ST elevations.

**Figure 2:**
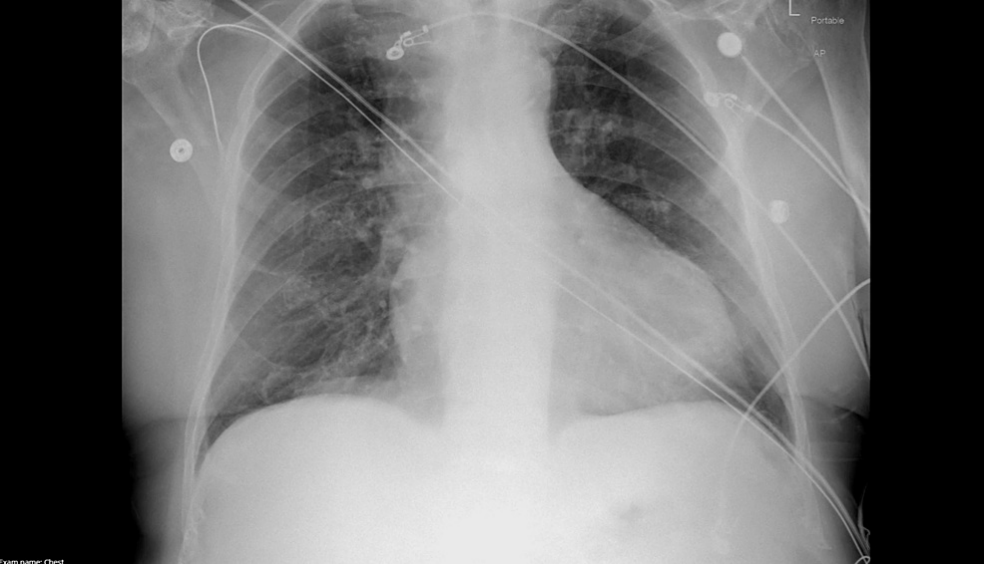
Initial chest X-ray significant for mild cardiomegaly with left ventricular prominence

His labs revealed a leukocytosis of 14.4x 10^9/L, hemoglobin of 9.4g/L, glucose of 215mg/dL, sodium of 134mmol/L, potassium 5.2mmol/L, blood urea nitrogen (BUN) 65mg/dL and serum creatinine (SCr) 2.6mg/dL (baseline BUN 40mg/dL and SCr 1.2mg/dL), high-sensitivity troponin of 40ng/L, lactate of 1.0mmol/L, B-type natriuretic peptide (BNP) of 430pg/mL, and C-reactive protein (CRP) of 17.8mg/L. Chest X-ray was only notable for mild cardiomegaly. The patient was admitted to the ICU for septic shock and acute kidney injury (Figures 1-2).

Septic shock resolved after one day of ICU services and his kidney function showed improvement with serum creatinine measuring 1.6mg/dL and BUN 46mg/dL. He was sent to receive an MRI of the right foot which demonstrated soft tissue ulceration on the medial aspect of the great toe at the level of the distal phalanx with findings of cellulitis, osteomyelitis, and septic arthritis involving the entire toe. CRP was measured at 17.8mg/L. Podiatry and infectious disease (ID) were consulted and wound cultures were collected. Initial wound cultures showed 4+ aerobic and anaerobic mixed bacteria including beta-lactamase-positive organisms. Podiatry recommended amputation given chronic ulceration of the right hallux and MRI findings. The patient was initially hesitant but finally agreed. Amputation of the right hallux was performed and intraoperative samples of the metatarsal head were taken for culture and pathology as well to ensure that clean margins were established. 

**Figure 3:**
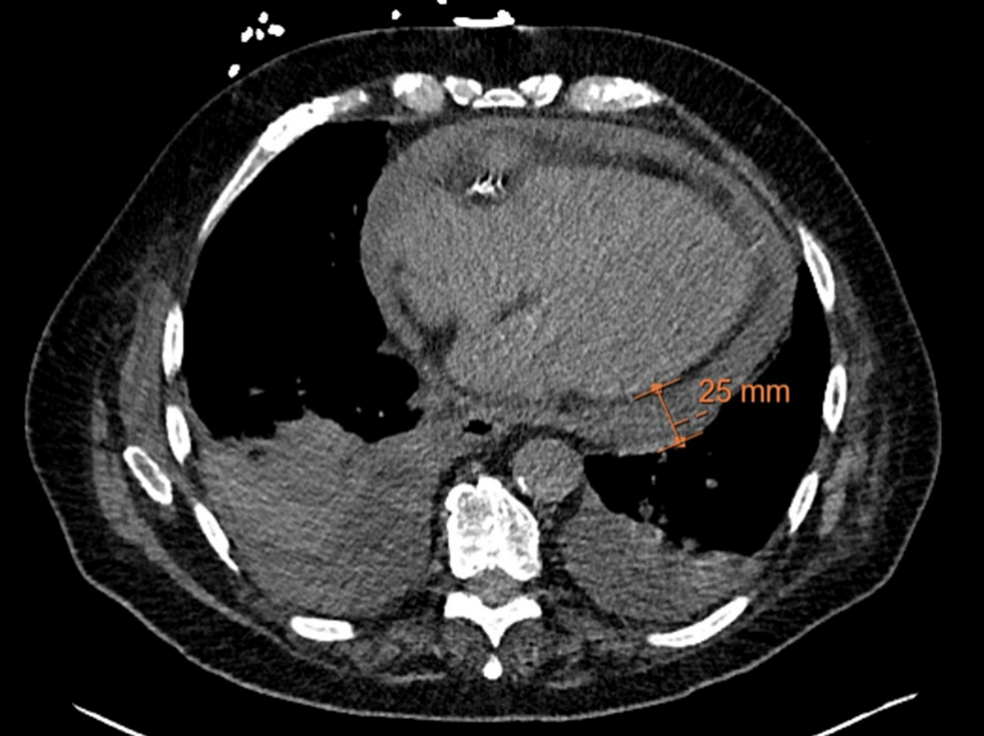
CT chest axial view demonstrating a moderate to large pericardial effusion and bilateral pleural effusions

**Figure 4:**
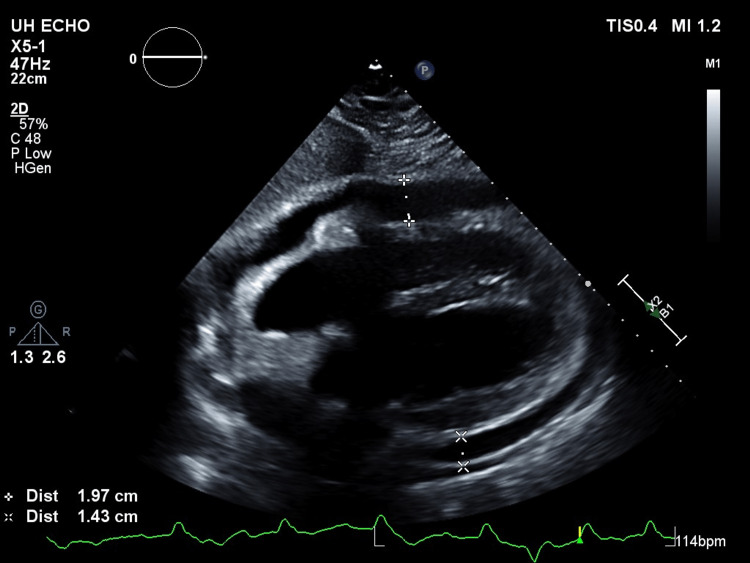
2-D echocardiographic parasternal long axis view of the heart with moderate to large circumferential pericardial effusion resulting in the beginnings of right ventricular collapse consistent with early cardiac tamponade physiology

**Figure 5:**
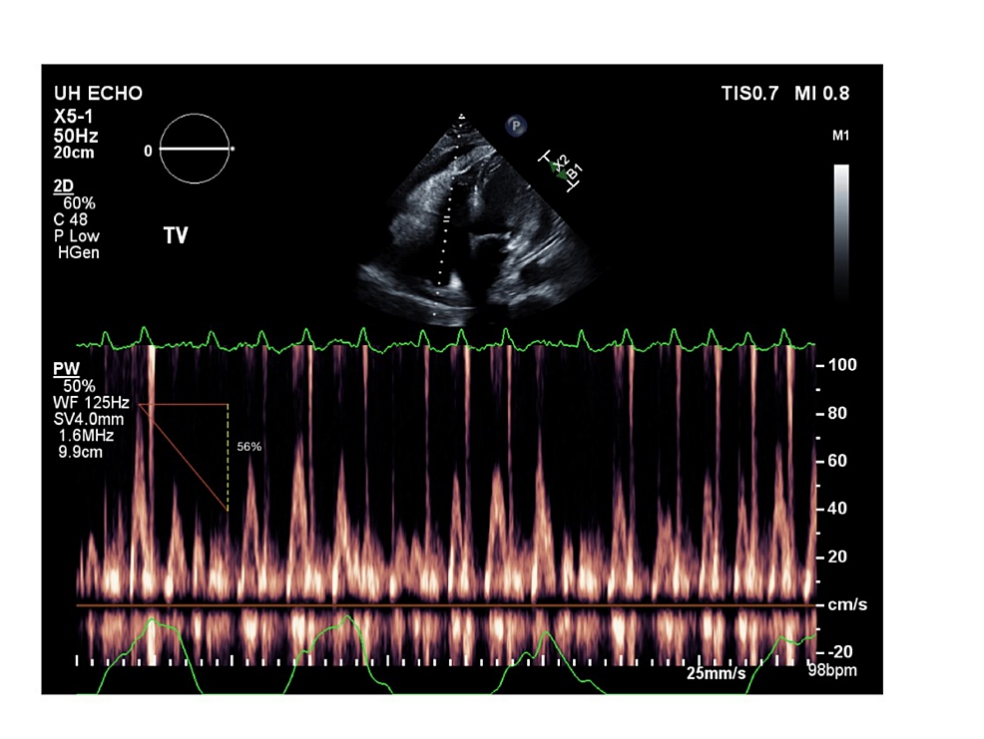
Continuous Doppler study showing the respiratory variation in tricuspid inflow velocity of 56% The peak E-wave velocity across the tricuspid valve will decrease by at least 40% in expiration compared to inspiration during cardiac tamponade [8].

Vancomycin and piperacillin-tazobactam were continued post-op while the ID team awaited intraoperative culture data which grew Enterococcus faecalis and methicillin-sensitive Staphylococcus aureus (MSSA). After four days of negative blood cultures, antibiotics were de-escalated to 3g IV ampicillin-sulbactam. The following day the patient endorsed worsening shortness of breath and was very somnolent on the exam. His WBC had increased from 14.8x 10^9/L the day prior to 17.8x 10^9/L. CRP was largely unchanged at 17.3mg/L. Given the increase in leukocytosis and worsening shortness of breath, a CT chest was ordered by ID. Ampicillin-sulbactam was continued. The CT chest demonstrated a moderate to large pericardial effusion (Figure 3). At this point, ampicillin-sulbactam was discontinued and vancomycin and piperacillin-tazobactam restarted. Echocardiogram performed the same day confirmed a moderate pericardial effusion without clear evidence of pericardial tamponade. The patient remained very lethargic and minimally arousable, however, vitals were within normal limits. He was also started on colchicine for pericarditis and a repeat echocardiogram the next day identified cardiac tamponade; the patient underwent an urgent pericardiocentesis (Figures 4-5). 

250mL of amber-colored pericardial fluid was drained which was sent to pathology for culture and cytology. A post pericardiocentesis echocardiogram showed significant improvement in the size of the effusion. However, the drain was left in place and the patient was sent to the ICU for closer monitoring. There was a total output of about 300mL of amber-colored fluid from the pericardial drain. The initial pericardial fluid analysis contained 1,300 WBC with 92% neutrophils, and 6,200 RBCs. The patient was continued on vancomycin and piperacillin-tazobactam.

The patient had improved mentation with a resolution of his leukocytosis. Pericardial fluid culture grew MSSA. Vancomycin/piperacillin-tazobactam was stopped and he was started on 2g IV nafcillin. However, given that the patient had grown Enterococcus faecalis as well as MSSA from his foot wound, the ID team replaced nafcillin with 1.5g IV ampicillin-sulbactam. He was discharged to a skilled nursing facility with four weeks of IV antibiotics and scheduled outpatient follow-up with cardiology and ID.

## Discussion

Pericardial effusion derived from bacterial infection of the pericardial space is an uncommon, but life-threatening, disease process. In the modern antibiotic era, the incidence of purulent pericarditis has decreased to one in 18,000 hospitalized patients; however, its progression can be very rapid, and half of all cases are diagnosed on autopsy [3]. This accelerated deterioration in a patient with purulent pericarditis can be secondary to hemodynamic collapse among others. The outer layer of the pericardium is stiff and inelastic, protecting the heart against pathogens by acting as a barrier. Its non-compliant structure allows the fibrous pericardium to also hinder severe dilation of the heart when volume within the chambers increases [9]. Consequently, this makes the pericardial sac unable to accommodate sudden increases in fluid collection within the pericardial space. Therefore, when the pressure within the pericardial space exceeds the pressure in any of the cardiac chambers during at least part of the cardiac cycle, tamponade physiology develops [10]. Interestingly, purulent pericarditis represents only about 8% of total cardiac tamponade cases, but of the cases of purulent pericarditis, almost half of them exhibit tamponade [11]. 

The most frequent presenting sign of purulent pericarditis is fever (85%) [3]. Only half of the reported cases will have ECG changes consistent with pericarditis and less than a third have an auscultatory friction rub or subjective chest pain [3,12]. Patients generally receive chest radiography while in the emergency department, which frequently reveals pulmonary infiltrates, pleural effusions, and mediastinal widening [12]. An echocardiogram is the most sensitive test and shows the presence of fluid in the pericardial cavity in almost all patients. However, the limitations of echocardiography are that it fails to differentiate purulent fluid collections from other causes of acute pericarditis [4]. Therefore, once the presence of a pericardial effusion has been identified, and there is suspicion of purulent pericarditis, a percutaneous pericardiocentesis should be quickly performed. This comes from the latest 2015 European Society of Cardiology (ESC) guidelines on the management of pericardial diseases that label pericardiocentesis for moderate to large effusions a Class I recommendation [13]. Ultimately, the removal of purulent pericardial fluid is a life-saving procedure, especially in cases of hemodynamic instability [7]. While there are many surgical approaches to removing the purulent effusion, pericardiocentesis is usually the first-line therapy to drain the excess fluid [13]. However, it should be noted that a potential complication of this approach is incomplete drainage which may increase the risk of subsequent constrictive pericarditis [14]. About 40% of patients will require surgical pericardiotomy after initial drainage with pericardiocentesis [15]. Therefore, it is strongly recommended to repeat imaging after initial pericardiocentesis to ensure complete and permanent drainage of the pericardial effusion [14]. 

The combination of vancomycin, fluoroquinolone, and third-generation cephalosporin should be included for critically ill patients [4,7]. After obtaining the pericardial fluid, samples should be sent for urgent microbiological staining, followed by culture sensitivities [16]. Depending on the results of these reports, empiric antibiotic therapy can be transitioned to four weeks of a bactericidal agent tailored to the cultured organism [4]. When considering follow-up of patients with purulent pericarditis, several parameters should be taken into account including the size and duration of pericardial effusion, the elevation of inflammatory markers, and the presence of symptoms [17]. However, in general, patients with a first-detected effusion should be followed every 1-2 weeks after the initial diagnosis and then after one month and six months [17,1]. 

## Conclusions

To summarize, our patient developed MSSA pericarditis with suspected hematogenous seeding from osteomyelitis of the right great toe. The original foci of infection were managed with amputation of the toe and IV antibiotics. Intraoperative bone cultures grew Enterococcus faecalis and MSSA, however, blood cultures were negative for the growth of either organism. Days later, echocardiography demonstrated a large pericardial effusion with findings suggestive of tamponade. The patient was treated with urgent pericardiocentesis and drain placement. Repeat echocardiography status post pericardial drain showed a significant reduction in pericardial effusion. The drain was removed once the output ceased. The patient was later discharged with baseline mentation, resolution of his cardiopulmonary symptoms, and scheduled IV antibiotics via a peripherally inserted central catheter (PICC) for four weeks.

Although bacterial pericarditis has become far less common, it should be high on the differential with new-onset, rapidly progressive cardiopulmonary symptoms, and mental status changes in the setting of a known focus of infection, as seen in our patient. If cardiac tamponade is identified by either clinical exam (a triad of muffled heart sounds, hypotension, and jugular vein distention) or echocardiography, urgent pericardiocentesis should be performed for if left unchecked, it can be rapidly fatal. Repeating the echocardiogram in the days following pericardiocentesis is recommended to check for resolution of the effusion or possible recurrence as seen in a subset of patients who require surgical pericardiotomy. 
